# Does Discrimination Explain High Risk of Depression among High-Income African American Men?

**DOI:** 10.3390/bs8040040

**Published:** 2018-04-19

**Authors:** Shervin Assari, Maryam Moghani Lankarani, Cleopatra Howard Caldwell

**Affiliations:** 1 Department of Psychiatry, University of Michigan Medical School, Ann Arbor, MI 48109-2029, USA; lankaranii@yahoo.com (M.M.L.); cleoc@umich.edu (C.H.C.); 2 Center for Research on Ethnicity, Culture, and Health (CRECH), University of Michigan School of Public Health, Ann Arbor, MI 48109-2029, USA; 3 Department of Health Behaviors and Health Education, University of Michigan School of Public Health, Ann Arbor, MI 48109-2029, USA

**Keywords:** African Americans, Blacks, gender, socioeconomic position, socioeconomic status, depression, discrimination

## Abstract

**Background:** Higher socioeconomic status is known to decrease the risk for poor mental health overall. However, African American males of higher socioeconomic status (SES) are at an increased risk for having a major depressive episode (MDE). It is not known whether perceived discrimination (PD) explains this risk. The current study used nationally representative data to explore the role of PD in explaining the association between high-SES and having MDE among African American men. **Methods:** The National Survey of American Life (NSAL), 2003, included 4461 American adults including 1271 African American men. SES indicators (i.e., household income, educational attainment, employment status, and marital status) were the independent variables. 12-month MDE measured using the Composite International Diagnostic Interview (CIDI) was the outcome. Age, gender, and region were the covariates. PD was the potential mediator. For data analysis, we used logistic regression. **Results:** Among African American men, household income was positively associated with odds of 12-month MDE. The positive association between household income and odds of MDE remained unchanged after adding PD to the model, suggesting that PD may not explain why high-income African American men are at a higher risk of MDE. **Conclusions:** Perceived discrimination does not explain the increased risk for depression among African American males of higher SES. Future research should explore the role of other potential mechanisms such as stress, coping, social isolation, and/or negative social interaction that may increase psychological costs of upward social mobility for African American males.

## 1. Background

In contrast to the mainstream theoretical [1] and empirical [2,3,4] work that emphasize the protective effects of socioeconomic status (SES) on population health, recent research has revealed a high risk of having major depressive episodes (MDE) in high-SES African American males [5,6,7]. This is consistent with a larger body of research that has shown smaller health return of SES for African Americans compared to Whites [8]. Education [6,8,9], income [8,10], and employment [11] have smaller protective effects on the physical and mental health for Whites than African Americans, as described by the *Minorities’ Diminished Return* Theory [12,13].

The seminal theoretical work by Link and Phelan [1], Mirowsky and Ross [14], and Marmot [2,3,4] do not explain the observed positive link between SES and depression in African Americans, particularly African American males [5,6,7], as these frameworks mostly conceptualize high SES as a protective factor for health. The positive link between SES and depression in African Americans is also in contrast to the literature on protective effects of SES on population health in Europe [15,16,17,18] and the U.S. [19,20,21,22].

*Minorities’ Diminished Return Theory* [12,13], suggesting that health gains from SES is smaller for African Americans compared to Whites, is also in contrast to multiple disadvantage [23,24] and multiple jeopardy [25,26,27] theories. All these models have traditionally conceptualized African Americans and other minority populations as vulnerable groups whose health is highly influenced by additional risk and protective factors (e.g., SES resources) [27]. In line with the *Minorities’ Diminished Return Theory*, extensive empirical research has shown that it is not African Americans, but Whites whose health is closely affected by SES [12,13,27].

Ironically, and in a counterintuitive pattern (i.e., inverse social gradient in mental health) [5,6,7], African Americans with high SES do not report better mental health than their low SES counterparts. In contrast, SES is positively associated with the risk of depression in African Americans, particularly African American males [5,6,7]. Although these patterns are in line with the *Minorities’ Diminished Return Theory* [12,13], it is still unknown why high SES sometimes operates as a risk factor for depression in African American males [2,3,4].

For at least two reasons, this study proposes perceived discrimination (PD) as an underlying mechanism for the positive association between SES and risk of depression in African American males. First, high SES is associated with higher, rather than lower, PD for African American males [5,7]. This is particularly relevant for depression in males, as some evidence has documented stronger effects of PD on depression and distress among males than females across ethnic groups including African Americans [28,29,30,31]. Second, PD reduces the health gains that high SES usually generates [32]. Research by Hudson et al. has shown an interaction between SES and PD, suggesting that high exposure to PD minimizes the health gain that is expected to follow high SES for African American men [32]. Research by Fuller Rowel has linked upward social mobility to poor health in African Americans [33,34]. This is also in line with the literature on John Henryism [35,36,37] and goal-striving stress [38,39,40,41] that are high among African American males and are positively linked to depression. This evidence collectively suggests that PD may potentially mediate the positive link between SES and depression in African American males.

### Aims

To extend our previous work in this area [7], and to uncover the underlying mechanism for the positive association between SES and major depressive episode (MDE) in African American males, this study tested the mediating effects of PD on the association between SES and MDE in African American males. To generate national results, data from a nationally representative study were used.

## 2. Methods

### 2.1. Design

National Survey of American Life (NSAL) is one of the most comprehensive and updated mental health surveys of Blacks in the U.S. The NSAL was conducted as a component of the Collaborative Psychiatric Epidemiology Surveys (CPES) by University of Michigan, Ann Arbor [42,43].

### 2.2. Participants and Sampling

NSAL used a national household probability sampling strategy of Black adults to generate representative statistics of Black Americans. The NSAL sample included 3570 African Americans. All participants were age 18 or older. The NSAL sample was composed of individuals who were residing in the U.S. at the time of the study. More detailed information regarding the NSAL sampling is available elsewhere [43]. The analytical sample of this study was 1271 African American men.

### 2.3. Process

In the NSAL, all interviews were all conducted in the English language. All face-to-face interviews used a computer-assisted personal interview (CAPI) format. CAPI is an interview model in which respondents use computers to provide answers to the questionnaire. CAPI improves data quality in long and complex surveys [44]. The NSAL response rate was about 71% for African Americans. Race and ethnicity were self-identified in the NSAL. African American was defined as Blacks in the absence of any ancestral ties to Caribbean countries.

### 2.4. Variables

The current analysis used data on demographic factors (i.e., age), SES indicators (i.e. household income, educational attainment, marital status, and employment status), and 12-month MDE.

Demographics. This study treated age as a continuous variable.

*Socioeconomic Status (SES)*. All measured using self-reported data, four SES indicators were entered to the current study: education attainment, household income, marital status, and employment status. Educational attainment (less than 12 years = 0; 12 years or more = 1), employment status (unemployed/not in labor market = 1; currently employed = 0), and marital status (currently married = 1; separated/divorced/never married/widow = 0) were all dichotomous variables. Household income was operationalized as a continuous measure and presented as income in increments of 1000 U.S. dollars.

*Major Depressive Episode (MDE)*. NSAL used the Composite International Diagnostic Interview (CIDI) to measure 12-month MDE, which reflects experience of a major episode of depression which lasted two weeks or more during the last 12 months. Main symptoms used to diagnose MDE in our study are depressed mood (guilt, worthlessness, sadness), and anhedonia (lack of interest or pleasure) for more than two weeks. MDE is not a score but a categorical diagnosis, so it is not based on a certain threshold. This diagnosis is based on the Diagnostic and Statistical Manual, Fourth Edition (DSM-IV) [45]. CIDI, a structured diagnostic interview schedule, is administered by lay interviewers (non-clinicians who have received proper training). CIDI diagnoses are based on the DSM-IV criteria [46]. CIDI based- diagnoses have acceptable reliability and validity, defined as concordance with blinded clinical diagnoses [45,46,47,48]. CIDI is widely used to measure MDE in African Americans [49,50,51].

*Perceived Discrimination*. Perception of daily racial discrimination was measured using the *perceived discrimination* instrument developed by Williams et al., 1997 [52]. The instrument accounts for perception of unfair treatment due to different characteristics. The measure does not refer to racism or discrimination, and it allows measurement of all types of discrimination due to age, disability, weight, gender, and race. This measure is not specific to racism and can be applied to all social groups. As this tool measures discrimination based on respondents’ perceptions, not observation of discriminatory events, we refer to discrimination as perceived discrimination.

The measure specifically focuses on daily racial discrimination that represents instances of discrimination that involve minor, rather than major, events and episodes that respondents recurrently face in their daily lives. Participants were asked about frequency of 10 types of unfair treatment that had occurred in their everyday lives. Response scale included “*never*” (coded as 0), “*less than once a year*” (coded as 1), “a few times a year” (coded as 2), “a few times a month” (coded as 3), “at least once a week” (coded as 4), and “*almost every day*” (coded as 5). A sum score for the 10 items were calculated, with a potential range from 0 to 50. The internal reliability of the measure was 0.93.

## 3. Ethical Considerations

The NSAL study protocol received Institutional Review Board (IRB) approval from University of Michigan, Ann Arbor (B03-00004038-R1). Respondents were financially compensated for participating in the study. Written informed consent was provided by all participants.

### Data Analysis

To accommodate the NSAL-Adults survey design, Stata 13.0 (Stata Corp., College Station, TX, USA) was used for data analysis. Design-based variance and standard errors re-were re-estimated using the Taylor series approximation techniques. As a result, means and percentages reported in this study were representative of the U.S. population. Weights were also used for all inferences, so our *p* values are based on comparison of weighted statistics.

Two sub-population survey logistic regressions models were estimated for multivariable data analysis. *Model 1* did not include PD. *Model 2* included PD. In our models, SES characteristics were the independent variables, MDE was the dependent variable, demographics and region were covariates, and PD was the mediator. Odds ratio (OR) and 95% confidence interval (CI) were reported. *p* values less than 0.05 was considered statistically significant.

## 4. Results

### 4.1. Descriptive Statistics

This study included 1271 African American men. Table 1 describes demographics, SES, PD, and 12-month MDE in the sample.

### 4.2. Effect of SES on MDE

Table 2 presents the summary of two logistic regressions with SES indicators as the independent variables and 12-month MDE as the dependent variable. Model 1 did not include PD. Model 2 also included PD. Based on Model 1, high income was a risk factor for high risk of 12 month MDE among African American men (Table 2).

### 4.3. Mediating Effect of PD on the Effect of SES on MDE

Table 2 Reports the results of Model 2 with 12-month MDE as the dependent variable, SES indicators as the main predictors, and PD as the potential mediator. The effects of all SES indicators remained unchanged in the second model, compared to the first model. That is, high income remained a significant risk factor for MDE among African American men (Table 2).

### 4.4. Summary of the Results (Mediation)

These results suggested that PD may not mediate the effect of income on odds of MDE among African American men.

## 5. Discussion

The current study was conducted to test whether PD explains the positive association between income and risk of MDE in male African Americans. The findings did not suggest that higher PD is why high-SES African American males are at a higher risk of MDE.

This is not the first study on the risks associated with high SES in African American males. It is also not the first study on the link between SES and depression by the intersection of race and gender [8,53]. In the NSAL-Adults data, high income was associated with an increased risk of MDE for African American men, net of other SES indicators [7]. In the NSAL-Adolescent data, high income is also shown to be a MDE risk factor for male African American youth [7]. In the NSAL-Adults data, education attainment was positively associated with suicidal ideation in Caribbean Black females [54]. Finally, in the Americans Changing Lives (ACL) study, in African American men, but not African American women, or Whites, high education attainment was a risk factor for an increased risk of depressive symptoms over time [6]. Among individuals above age 50, the protective effects of SES (education and income) were mostly absent for African American men [8]. Although these studies have documented diminished gain or even increased risk of depression among high-SES African American males, the role of PD in this relationship in still unclear.

This study is, however, one of the first to explore the role of PD in explaining high risk of MDE in high-SES African American males. Previous theoretical work has proposed PD as a potential underlying mechanism for Blacks’ diminished return of SES (i.e., low health gain of African American from high SES) [12,13]. This is particularly plausible as among African Americans, males report more PD and are more vulnerable to PD compared to females [29,30]. Among African Americans, PD increases, rather than decreases, as SES increases [32]. Our study, however, failed to confirm that high PD explains higher risk of MDE in high-SES African American males. 

These findings should not be considered against the overall protective effects of SES on health [55,56,57,58,59,60,61]. They are indicating considerable population heterogeneity in the effects of SES [32,62,63]. Populations differ widely in their opportunities to gain health benefits from SES resources [12,13]. As a result, scholars may shift their focus from overall health effects of SES to mechanisms for population differences in gaining health from SES.

In this study, PD was a risk factor for MDE, regardless of covariates. Literature is well-established that PD reduces both physical and mental health [64,65,66,67,68,69,70,71,72]. PD triggers a wide range of negative emotions and cognitions such as sadness, anger, worries, withdrawal, and loneliness [73]. In addition to depression [29], PD also increases risk of anxiety [30] and distress [28]. PD increases hyper-vigilance [74] which increases attention toward discrimination and attribution of ambiguous exposures and cues to discrimination [75]. Individuals who perceive high PD rate their social interactions as harassing [73], often results in social isolation [73]. PD also increases health risk behaviors such as smoking [76], drinking [77], drug use [78,79], and suicide [80].

### 5.1. Theoretical and Policy Implications

These results may have implications for theory as well as policy. Most current conceptual frameworks (e.g., fundamental cause theory (FCT)) have exclusively focused on the protective effects of high SES [1,58]. Being informed by these theories, most scholars have focused on the protective effects of high SES for population health [58,59,60]. Although the overall protective effects of SES are well-established and unquestionable [15,16,17,18,19,20,21], high SES does not mean health benefits for all social groups [5,6,7]. For minorities who are constantly struggling with societal barriers, and for racial and ethnic groups that are differently treated by society, high SES is not all about health protection [12,13,33,34]. This is particularly the case for social groups that experience higher PD as they climb the social ladder [81]. In these situations, high SES may also mean mental health vulnerability for racial and ethnic minorities. In response, policy solutions should go beyond equalizing SES and focus on addressing PD in the lives of racial and ethnic minorities.

### 5.2. Future Directions for Research

As the current study failed to show PD as an underlying mechanism for high risk of MDE for high SES Black men, other mechanisms should be explored in future research. Several mechanisms are involved in the link between SES and health. SES resources enable individuals to escape harmful exposures [1,58]. Individuals from higher SES positions are more likely to be in a high pay, low stress occupation that minimizes their exposure to environmental risk factors [63,82,83]. High SES also minimizes the consequences of risk factors when they are faced [59,60]. SES indicators, such as education, reflect population human capital [84]. SES resources increase population access to materialistic (e.g., healthy food, safe living environment, and income) as well as non-materialistic (e.g., social network and social support) resources [33,34]. SES resources enhance access to increasingly powerful human connections in the social network [34]. High-SES increases health-promoting behaviors such as healthy diet, exercise, and high-quality sleep and reduces health risk behaviors such as substance use [85]. Individuals with higher SES have better health literacy and better access to health care, as well as a better health care insurance coverage due to their employment [1,58,59,60]. SES resources enhance a wide range of psychosocial assets, such as emotion regulation, coping, sense of agency, and sense of mastery [86], all of which can buffer the effects of stressors [87,88]. Variation in all these mechanisms should be explored across various race by gender sub-groups [9,89,90,91,92,93,94].

### 5.3. Limitations

Our study is not free of limitations. First, SES measures were not ideal. We defined income as continuous dollar amounts, which does not account for the differential value of the same income based on regional/geographic location of residence. For example, one thousand dollars of additional income does not similarly change financial and/or social statuses of individuals residing in New York and Detroit. Employment status was coded as a dichotomous variable (unemployed/not in labor market = 1; currently employed = 0). This over-simplified measure fails to capture underemployment, as well as part-time versus full-time employment. Income was also treated as a continuous measure. Educational attainment is measured with a dichotomous variable (i.e., less than 12 years = 0; 12 years or more = 1). This categorization ignores differences in the educational level of individuals with a high school diploma from people who have achieved a doctorate degree. We used dichotomous measures of education and employment to reduce the co-linearity between various indicators of SES, particularly with income. Second, the study may have omitted variables such as receiving treatment for depression and other psychiatric disorders. We did not control for a wide range of relevant covariates, such as health behaviors, physical health, and history of depression diagnosis by a health care provider. Despite these limitations, using a nationally representative sample with large sample size is a strength of our study.

## 6. Conclusions

The current study failed to show PD as a mediator for high risk of MDE in high-SES African American males. Factors other than PD may be studied to understand why high-SES African American males are at high risk of depression. Future research should examine the role of additional psychological and social costs of upward social mobility, stress (e.g., goal-striving stress), negative social interaction, coping, social isolation, or institutional racism to explain the high risk of MDE in African American males. 

**Findings:** NSAL is funded by the National Institute of Mental Health (NIMH; U01-MH57716). Other funding includes support from the Office of Behavioral and Social Science Research (OBSSR) at the National Institutes of Health (NIH). University of Michigan (UM) also provided some funding for the NSAL.

## Figures and Tables

**Table 1:** Summary of descriptive statistics in African American men.

	African American Men	
	**Mean**	**95% CI**
Age	41.76	40.44–43.09
Income (1000 USD)	4.18	3.79–4.57
Perceived Discrimination (PD)	13.76	12.79–14.73
	**%**	**95% CI**
Region		
Northeast	16.04	13.08–19.53
Midwest	16.15	13.08–19.78
South	57.20	51.73–62.50
West	10.61	8.43–13.26
Socioeconomic status		
Education		
11 Years or less	22.65	19.54–26.08
12 Years or more	77.35	73.92–80.46
Unemployment		
Employed/Not In Labor Market	91.08	88.71–92.99
Unemployment	8.92	7.01–11.29
Marital Status		
Married	50.22	46.84–53.59
Unmarried	49.78	46.41–53.16
12 Month Major Depressive Episode		
No	95.29	93.72–96.48
Yes	4.71	3.52–6.28

**Table 2:** Logistic regression on the effects of socioeconomic resources and perceived discrimination (PD) on risk of 12-month major depressive episode (MDE).

	African American Men			
	OR	95% CI	OR	95% CI
Age	0.99	0.97–1.00	1.00	0.98–1.01
Region ^a^				
Midwest	3.99 *	1.15–13.87	3.16 *	1.09–9.10
South	1.29	0.49–3.39	1.34	0.54–3.33
West	1.90	0.43–8.43	1.70	0.36–7.98
Income (USD 1000)	1.10 ^#^	0.99–1.21	1.10 *	1.00–1.20
Education (12 Years or more)	0.60	0.29–1.25	0.59	0.28–1.23
Unemployed	1.63	0.61–4.34	1.61	0.64–4.06
Married	0.35 *	0.14–0.92	0.33 *	0.12–0.91
PD			1.06 ***	1.03–1.09
Intercept	0.06 ***	0.02–0.24	0.02 ***	0.00–0.09

Source: National Survey of American Life (NSAL); Outcome: 12 Month Major Depressive Episode; OR odds ratio, CI confidence interval; ^#^ *p* < 0.1; * *p* < 0.05; *** *p* < 0.001; ^a^ Reference category: Northeast.
